# Measuring intangible cultural heritage image: A scale development

**DOI:** 10.1371/journal.pone.0299088

**Published:** 2024-06-03

**Authors:** Yuqing Liu, Ye Li, Wenjie Tao, Qingsheng Wang

**Affiliations:** 1 School of Management, Tianjin University of Commerce, Tianjin, China; 2 College of Education, Tianjin Normal University, Tianjin, China; Universiti Kebangsaan Malaysia, MALAYSIA

## Abstract

Although an increasing number of studies have examined issues relating to the preservation and development of intangible cultural heritage (ICH), there has been limited research on how tourists perceive ICH. Moreover, UNESCO asserts that the concept of “authenticity” is not applicable to ICH, and so far, no valid instrument for measuring tourists’ subjective perceptions of ICH has been developed, even though their perceptions play a very important role in the preservation and development of ICH. Therefore, this paper aims to develop a measurement scale for ICH image, using both qualitative and quantitative research methods. Participant observation, semi-structured in-depth interviews, secondary data collection, and a literature review were conducted to generate the initial scale items, and then the main surveys were conducted to collect data for the model tests. Four dimensions were extracted by exploratory factor analysis: transmission, localization, vitality, and association. The reliability and validity of the measurement model were demonstrated through confirmatory factor analysis. We further determined that the transmission, vitality, and association of ICH image have a positive impact on tourists’ revisit intention. The paper highlights the crucial role of ICH image in sustainable tourism development. The theoretical and managerial implications of the study are discussed, followed by suggestions for future research.

## Introduction

Cultural heritage is an important aspect of our shared global historical legacy. Intangible cultural heritage (ICH) is a component of cultural heritage that is embodied in practices, representations, and expressions, as well as in related objects and spaces, and is constantly re-created by communities and groups [1]. ICH is important not just as a cultural manifestation but also as a way of transmitting knowledge and skills from generation to generation [2]. Through interactions with nature and history, ICH provides a sense of identity and continuity, in addition to promoting cultural diversity and human creativity [3, 4]. Since tourists wish to experience different cultures and enjoy a variety of performing arts, social festivals, local cuisines, and traditional handicrafts, ICH has the potential to transform places into tourism destinations [5]. Heritage tourism not only contributes to economic growth in culture-rich destinations but can also help to safeguard and develop ICH [6]. However, as standardization and mass production are convenient ways to satisfy the demand for ICH-based tourism, there is a danger that tourism may lead to the over-commodification of ICH and ultimately to cultural deterioration and even destruction and disappearance.

Researchers employ the concept of authenticity to investigate how well heritage is preserved [7]. Although ICH is often exhibited in the form of tangible objects, artifacts, or places, it is distinct from tangible heritage in that ICH is “living” [8]. Therefore, according to UNESCO, “because intangible heritage is constantly re-created, the concept of “authenticity” applied to World Heritage properties cannot be used for ICH” [9]. There is a gap in the tourism studies literature regarding how to measure tourists’ perceptions of ICH. Given the importance of ICH to tourism and the potentially negative impact of tourism development on ICH, this paper proposes to fill this gap by developing a measurement scale for ICH image, thus contributing to proper ICH management [10]. We suggest that the “ICH image” can be used to understand how tourists perceive a specific ICH during the process of tourism development. “Image” is a widely used concept in tourism studies found in concepts such as “destination image,” “place image,” “tourism organization image,” “tourism product image,” and “tourist attraction image.” An “image” is an individual’s overall beliefs, ideas, and impressions of an object [11]—in this study, the object is ICH. These beliefs, ideas, and impressions are derived from the object’s attributes. In the case of tourism experiences, there is a high degree of variation among the objects involved (e.g., [11–13]), meaning that it is necessary to determine the attributes of a specific ICH to conduct a valid and reliable image measurement. An ICH image measurement scale would need to be specially designed for this purpose [14]; the image scales in the existing tourism studies literature are not suitable for measuring ICH image. Furthermore, in spite of the importance of tourist revisit intention to the success of tourist destinations, previous studies have mainly focused on the role of ICH in attracting new tourists [15] but not on how ICH image affects revisit intention. Therefore, this paper aims to raise awareness of tourists’ perceptions of ICH by developing a measurement scale to capture perceived ICH image and by exploring how ICH image affects tourists’ revisit intention.

From a theoretical perspective, this study develops a scale for ICH image measurement based on an analysis of the ICH’s attributes, thus deepening our understanding of individual cognitive perceptions of ICH. We reveal that ICH image is a multidimensional second-order construct with a structure that can be represented well by our measurement scale. Our study contributes to the literature by examining the relationship between ICH image and tourist revisit intention to explain tourist behaviors in ICH-based tourism. From a practical perspective, the ICH image has a role to play in striking a balance between tourism development and ICH sustainability in an era when the UNESCO’s Lists of Intangible Cultural Heritage seem to promise the tourism market that the ICHs on the list are worth visiting [6]. The original purpose of UNESCO’s Lists of Intangible Cultural Heritage was to maintain the sustainability of ICH in the face of growing globalization, despite the risk that too many visitors could bring cultural change or even deterioration, disappearance, and destruction due to over-commodification. Given that over-commodification is likely to lead to negative images of an ICH [7], measuring ICH image can help ICH management organizations check for over-commodification in ICH-based tourism and take adequate steps to guarantee the balance between tourism development and cultural preservation.

The remainder of this manuscript is organized as follows. Next, the existing literature relevant to ICH, ICH in tourism development, and ICH image is reviewed. Then, following the scale development procedure proposed by Churchill [16], we specify the domain of the ICH image, generate initial items, and collect data. This is followed by the data analysis, which is used to purify the measurement items and develop a reliable and valid scale. The results of the data analysis are then discussed. Finally, the conclusions and theoretical and managerial implications are discussed, followed by an account of the study’s limitations and opportunities for future research.

## Literature review

### Intangible cultural heritage

UNESCO has included ICH in heritage concerns since the 1990s. According to UNESCO’s convention for safeguarding ICH, all sorts of practices, knowledge, and skills transmitted from generation to generation, patterns of representation and expression, as well as the associated instruments, objects, artifacts, and cultural sites, as well as related communities, groups, and some individuals, are recognized as part of ICH [3]. ICH plays a vital role in protecting cultural diversity and increasing social cohesion and contributes to individual self-identity and cultural identity [1, 3]. ICH encourages intercultural dialogue among different communities and promotes respect for other ways of life.

UNESCO proposes five broad domains under which ICH can be classified: (1) oral traditions and expressions, (2) performing arts, (3) social practices, rituals, and festive events, (4) knowledge and practices concerning nature and the universe, and (5) traditional craftsmanship [3]. These five domains are tightly linked to everyday life, which includes a wide range of cultural elements [17]. The boundaries between domains are not constant, and the list of domains may be inclusive but not exclusive [9]. For example, festivals are a complex expression of ICH and may involve traditional music, dance, and displays of craftsmanship, as well as ritual and ceremonial practices.

ICH differs from tangible heritage in several ways. First, the most important characteristic of ICH is that it is community-based and has never left the lifestyle and practice of the groups or individuals that create, maintain, and transmit it [18]. Second, ICH is inclusive, which means that a range of different cultural expressions that may evolve in response to totally distinct environments are included in ICH [17]. Third, ICH is simultaneously traditional, contemporary, and living, and represents the “living” memory of a particular community [19]. Fourth, the conservation of ICH is more difficult than that of tangible heritage since ICH is preserved through generational inheritance, not in the restoration and maintenance of ancient remains [20].

### Intangible cultural heritage in tourism development

ICH and tourism development are frequently discussed in conjunction with each other [21]. Heritage tourism includes diverse aspects, such as cultural and inherent history, indigenous stories and rituals, engaging with places, artifacts, and activities that purport to authentically represent the past [22]. Thus, heritage tourism can be defined as “the explicit and voluntary contact that tourists have, away from their normal places of residence, with cultural heritage through the visit or consumption of heritage goods and services” [23]. However, contrary to UNESCO’s definition of ICH as a process, this definition regards cultural heritage as a product rather than a process. ICH tourism can be defined as a trip-based experience of places, crafts, and activities that undoubtedly represent community stories and people in the past and present [24]. The main branches of research on ICH tourism include ICH resource planning and sustainable development, the impact of ICH tourism, and ICH destination marketing and tourist behavior [25]. These branches of research are closely related—for example, understanding tourists’ perceptions of ICH will help determine how to balance tourism development and ICH preservation [6, 10]. Sustainable development of ICH as tourism attractions can increase employment opportunities, contribute to poverty alleviation, reduce the migration of young people to other areas, and nurture a sense of community pride [2]. Moreover, tourism helps fund the preservation and enhancement of ICH because tourism industry revenues can be channeled back into maintaining the long-term survival of ICH [4, 20]. The challenge for the tourism sector is to identify, protect, and safeguard ICH through tourism development. Tourism policies should ensure that artistic, archaeological, and cultural heritage is protected and passed on to future generations. There are many types of tourism activities that contribute to ICH preservation and sustainability at the destination, such as community-based tourism and cultural tourism initiatives [26]. The UNWTO recommends that local communities should be active decision-makers in tourism development, as opposed to passive recipients of tourism [2]. Research suggests that ICH should be performed not for the consumption of tourists but by the communities for their own cultural purposes [5]. For various communities, because skills and knowledge that cannot be duplicated by others and the continuity of these ICH depend on the transmission to successive generations, uncontrolled or overly rapid tourism development can disrupt established transmission mechanisms. Moreover, when a community begins to see itself as a cultural attraction, the members of the group may start treating themselves as representatives of an ethnic way of life, leading to the ICH becoming frozen or “museumized” [27]. In planning tourism activities, care should be taken to allow ICH to survive and flourish without causing the culture to stagnate or become a standardized commodity.

The question of whether commodification can cause cultural heritage to lose its value or become meaningless has long been examined in tourism research. The UNWTO argues that an ICH needs some level of commodification to make its cultural value understandable to tourists [2], and that cultural expressions of heritage need to be transformed into commodity products that can be consumed by tourists [5]. Although commodification is considered a necessary step for cultural heritage to engage with the tourism market, the commercialization process can cause substantial harm to cultural heritage. The commercialization of heritage may entail simplifying living culture to increase transaction amounts [17]. Over-commodification can also lead to remote and mysterious cultures losing their appeal, because commercialization makes them become more like the tourist’s own culture, with fewer differences or distinctions, so that they are no longer seen as “primitive” [7].

For tourism practitioners and researchers, the concept of the authenticity of tourism destinations, sites, events, cultures, and experiences is crucial for heritage tourism planning, marketing, and management [28]. Authenticity is considered an initial, prevalent value and driving force motivating tourists to go to different places and experience other cultures [29], especially in the era of postmodernism [30]. In heritage, authenticity means that cultural value is defined truthfully and that genuine testimony to cultural value is guaranteed in signifying a traditional culture and origin [31]. Although many studies suggest that the quality of heritage tourism is improved by perceived authenticity [e.g., 32], whether authenticity should be applied to ICH remains an open question [33]. UNESCO states explicitly that the concepts of “authenticity” or “integrity” applied to tangible heritage should not be used for ICH because of its “living” character [9, 34]. Moreover, Lixinski asserts that authenticity establishes control over the uses and meanings of heritage, and that pursuing ICH authenticity can lead to dangerous commodification, potentially restricting a living, constantly evolving culture [5]. However, if ICH tourism is to be developed, a way of depicting the characteristics and attributes of ICH is needed, and adequate management of ICH tourism requires an understanding of tourists’ perceptions of ICH to strike a balance between commodification and cultural protection [10]. As authenticity is not an appropriate concept for assessing ICH, it needs to be assessed by subjective criteria [35]. Therefore, we propose developing the ICH image construct to explicate and measure the subjective cognition of ICH in tourism development.

### Intangible cultural heritage image

The importance of images is well recognized by tourism scholars, as they influence tourists’ subjective perceptions, decision-making, and consequent behaviors [11, 36]. An image can be defined as an overall impression of an object, which could be a tourism product, attraction, accommodation, tourism supplier, place, destination, or country. Destination images are the main research focus in the tourism research field [37]. Early studies characterized destination images as encompassing tourists’ mental perceptions of a destination; a recent study gave a more precise definition: “a voluntary, multisensory, primarily picture-like, qualia-arousing, conscious, and quasi-perceptual mental experience held by tourists about a destination” [38]. These definitions reveal the complexity of images, which can be analyzed using different approaches [36]. There are three main approaches to analyzing images in the research literature: a three-dimensional model (including attribute-holistic, common-unique, and functional-psychological) [39], a three-component model (comprising cognitive image, affective image, and conative image) [40], and a long tail or core–periphery structure [41, 42]. In our exploration of ICH image, we draw on the most commonly used three-component model. We also concentrate on cognitive image of ICH because cognitive images have been the focus of previous studies and can be applied to understand an object’s attributes [14, 43]. To properly manage ICH tourism, information about tourists’ perceptions of ICH is necessary [10]. From a cognitive perspective, ICH image is relevant to a person’s rational beliefs and knowledge about an ICH’s attributes; this opens up a window for us to understand tourists’ perceptions.

As the concept of “image” can be applied to many objects in the tourism context, quite a few studies have investigated “heritage image,” which refers to the tourist’s impression of cultural heritage sites and can have a positive impact on experiential quality and satisfaction and behavioral intention [44]. Saeedi et al. revealed that heritage image positively impacts the common image of the heritage destination, thus contributing to destination branding [45]. However, few studies have explored the measurement of heritage images [44], which gives rise to trouble understanding tourist perceptions. The extant research either adjusts the measurement of destination images or chooses some features or attributes of tangible heritage to measure the heritage image. We suggest that these measurement scales are not suitable for measuring ICH image and that a new scale needs to be constructed. This, in itself, is an important research focus for several reasons. First, an image measurement scale should be context-specific [14], and ICH have their own characteristics, which are community-based, inclusive, and “living,” [1, 17–19]. To illustrate the unique characteristics of ICH, all of these characteristics need to be reflected in the measurement of ICH image. Second, the tourism destination attributes used in the cognitive approach to measuring destination image are difficult to adapt to measuring ICH image. The attributes of tourism destinations used in destination cognitive image measurement [46, 47], such as climate, safety, cleanness, accommodation facilities, restaurants, and transportation, are unrelated to ICH; therefore, a new ICH image scale with better validity and reliability is required. Third, ICH differs from tangible heritage with respect to safeguarding [20], meaning that authenticity is not an appropriate concept for describing ICH during tourism development. Tangible heritage is a constant legacy, and preserving its original form is important for heritage tourism, whereas ICH is constantly re-created, and keeping a prestigious image is crucial for sustainable tourism development; therefore, image measurement scales previously developed in the tangible heritage context fail to capture the constantly re-created feature of ICH. Finally, creating image and branding ICH are considered useful for destination marketing, and these impacts are usually positive [25], which means that ICH and tourism have a mutually beneficial relationship. An effective way to safeguard ICH is to preserve the uniqueness and attractiveness of its image during the process of tourism development. Although many studies have revealed this mutual relationship, there is a lack of studies exploring what ICH image is and how to measure it. From this perspective, there is a need to develop an ICH image measurement scale. To capture the characteristics and attributes of ICH and avoid potential bias from using only one method, we used different investigation methods—including semi-structured interviews, participant observation, and secondary data collection—to develop the initial items.

### Methodology

The scale development procedure proposed by Churchill [16] includes five major steps: (1) identifying the image domain, (2) initial item generation, (3) measurement item purification, (4) data collection, and (5) reliability and validity verification of the proposed measurement scale. We followed this procedure closely to develop the measurement scale for ICH image. We obtained written Ethical Approval from the Ethics Committee of Tianjin Normal University for this study.

### Construct domain

Identifying the domain of the construct means answering the question of what should be included in the definition and what should not [16]. Through the literature review, we found that there is a general consensus that the “image” is the overall impression of an object, and that disagreements about this concept concern how these impressions are formed [38]. Moreover, we demonstrated that the cognitive approach in image research is more appropriate than the affective approach in developing a measurement scale for ICH image. Thus, we follow previous studies and define ICH image as an individual’s overall impression of ICH. As “image” is a multidimensional construct [11], the key point of scale development for ICH image is not only to find measurement items but also to reveal the construct dimensions.

### Item generation

To generate the initial items, we adopted four methods: semi-structured in-depth interviews, participant observation, secondary data collection, and a literature review. For the semi-structured in-depth interviews, the interviewees were recruited using purposive sampling. Based on sustainable heritage tourism development principles and stakeholder’s theory, governments play a crucial role in ICH protection and development [48], which has a significant impact on tourists’ perceptions of ICH [49], five specialists (four males and one female, from 30 to 50 years old) in cultural heritage management were recruited. They are all staff members at the Government Bureau of Culture and Tourism (district level) and are responsible for managing ICH protection and development within the districts of Tianjin. These semi-structured, in-depth interviews were conducted within the context of a research project on the merging of culture and tourism in Tianjin. To gain insights into the development and protection of ICH and their impacts, four main questions were asked: “Which examples of ICH are present here? What is their current condition?”, “Has the ICH been developed for tourism? If yes, what has been the effect of the development?”, “How can ICH be protected during tourism development?”, and “Are any ICH products (or experiences) being sold to tourists? How do tourists seem to feel about them?”). All interviews lasted 30–45 minutes, and the key points were recorded. To avoid coding bias, two researchers coded the interview data independently, and if there were different opinions among the coders, group discussions were held to reach a final decision. After open coding to conceptualize the interview data and axis coding to group the concepts, nine categories emerged (see Table 1), which can be compiled as nine test items.

**Table 1:** Coding results.

Number	Categories	Open Coding	Examples
1	Well protected	preservation, keeping inheritance, protective measures, unchanged	The inheritor emphasizes the preservation of the original flavor. (preservation)
2	Clear process of transmission	generational inheritance, introduction of inheritors	How the ICH is passed down between generations, is there any interruption, and what is the reason for it are clear. (generational inheritance)
3	Related to the local environment	related to the local climate, local things, receives nourishment from the place	The emergence of this production method is related to the local climate. (related to local climate)
4	Artistic style	festive, vivid, exaggerated, popular	This is very festive. (festive)
5	Diverse forms of expression	variety, different strengths, innovation, different contents	I feel like I have not experienced this (ICH) before—it is very different. (variety)
6	Significant in cultural inheritance	life history, cultural history, inheritance of experiences	From this ICH, it can be seen how people lived at that time. (life history)
7	Aesthetic value	exquisite, delicate, aesthetic meaning	This is really exquisite. (exquisite)
8	Worth visiting personally	on-site experience, personal experience	This is a type of local culture, and if you’re not here, you won’t feel it. (on-site experience)
9	Significant impact on history	important former custom, former belief	People in the past attached great importance to this, and had to put up New Year pictures during the Chinese New Year. (important former custom)

For the participant observation, we visited several famous and important ICH in Tianjin: Hui Minority Heavy Blade Wushu, Hollow Wood Carving, Huifuyuan Handcraft Fabric Art, Ear Hole Fried Cake, Zhimeizhai Braised Beef Making, and Yidecheng Smelling Medicine. We recorded the visiting process, identified the attributes of these ICH, and then generalized the attributes to obtain four test items, including: “Local people identify with the ICH”, “The ICH reflects the local lifestyle”, “The ICH is transmitted from generation to generation”, and “The inheritor (performer) of the ICH is highly skilled”.

To address the problem of our semi-structured in-depth interviews and participant observation being concentrated in one area, we collected secondary data on ICH from UNESCO’s Lists of Intangible Cultural Heritage. These include descriptions of the ICH and tourist reviews of them. These ICH are taken from the domains of dramatic performance, social tradition, ritual, festive events, special knowledge and natural practices, and conventional craftsmanship (e.g., making Xuan paper, Dragon Boat Festival, Peking Opera, etc., which are all on China’s state-level ICH list). After the coding process, another seven measurement items referred to the following: “The ICH has collectible value”, “The ICH has special artistic traits of nations”, “The ICH is preserved in good condition”, “Beliefs can be placed on the ICH”, “The ICH satisfies my cultural curiosity”, “The ICH helps me understand cultural inheritance”, and “I interacted with the inheritors (performers) of the ICH”.

Finally, we reviewed destination image studies and cultural heritage authenticity studies [50–53] to compare the measurement dimensions and enrich the measurement pool further, then added another five test items, which are “I immerse in the process of appreciating the ICH”, “The ICH contributes to my personal development”, “The ICH is related to my life”, “The ICH makes me feel a sense of connection with the relevant history”, and “The ICH makes me feel a sense of connection with human civilization”. In total, we derived 25 items to formulate the ICH image scale.

### Pilot test and measures purification

A pilot test was conducted to assess the quality of the initial measurement items. A convenience sampling approach was adopted to recruit participants for interviews to test the ICH image scale, and 35 valid responses were received. All responses were from college students in the same class, with similar demographic characteristics (68.6% women, 31.4% men). After content validity analysis and further interviews with the respondents to explore why some items were not appropriate for the ICH image measurement, three items were deleted. The most common reason was that the respondents were unable to distinguish the difference between two items, as in the case of the items “The ICH is well protected” and “The ICH is preserved in good condition” (we deleted the latter). The revised ICH image scale included 22 items.

### Data collection

We conducted two formal surveys, one for exploratory factor analysis (EFA) and the other for confirmatory factor analysis (CFA). Survey 1 (for EFA) included two parts: one was the ICH image scale, and the other was demographic characteristics (gender, age, level of education, profession, and income). Survey 2 (for CFA) included three parts: the revised ICH image scale, which contained 12 items after exploratory factor analysis; demographic characteristics; and a scale to measure tourist revisit intention developed by Kim [54], which includes three items (“I would like to revisit this ICH,” “I plan to revisit this ICH,” and “I will make an effort to revisit this ICH within a year”). The 22 items of the ICH scale and the three items on the revisit intension scale were scored on a 7-point Likert scale ranging from 1 (strongly agree) to 7 (strongly disagree).

The survey sampling was conducted by a professional information collection company that utilized two technologies to increase response quality: one was the time taken to answer the questions, and the other was IP address checking. If two responses had the same IP address (within one survey or between Surveys 1 and 2), they would both be deleted. In addition, if the respondent took less than four seconds to answer each item on the questionnaire, the response would be deleted.

We set two filter questions to further improve the response quality. The first required respondents to indicate which of six different ICH they had had contact with (by watching, learning, experiencing, and/or buying); if they could select none of these, they were asked to select “other” and to specify what the other ICH was. The six ICH were: Chinese shadow puppetry; *Errenzhuan* (northeast song-and-dance duets); Suzhou embroidery; Chinese paper cutting; Yangliuqing New Year paintings; and Chinese herbal tea, which are all on UNESCO’s Representative List of the ICH of Humanity. The second question required respondents to describe their own experiences with the ICH they selected. For the first question, if the respondents chose “other” but then described something that is not an ICH, the response would be deleted. For the second question, if the description was not relevant to the ICH they chose, the response would be deleted. After careful screening, we obtained 337 valid responses in Survey 1 and 380 valid responses in Survey 2 (see Table 2). The item and sample ratio was higher than the ratio of 1:10 required by Tabachnick and Fidell [55].

**Table 2:** Demographic profile of the sample in Surveys 1 and 2.

Profile category	Survey 1	Survey 2
**Gender**		
Male	48.1%	55.0%
Female	51.9%	45.0%
**Age**		
25 and below	22.6%	27.4%
26–35	57.2%	46.8%
36–45	19.0%	18.7%
46–55	1.2%	6.8%
56 and above	0%	0.3%
**Education**		
Up to high school	11.3%	16.6%
College degree	27.3%	36.3%
Bachelor’s degree	59.9%	43.9%
Postgraduate degree and above	1.5%	3.2%
**Income per month**		
RMB 1–5,000	20.2%	18.4%
RMB 5,001–8,000	58.2%	53.5%
RMB 8,001 and above	14.0%	15.5%
No income	6.2%	10.5%
Prefer not to say	1.4%	2.1%
**Profession**		
Student	9.2%	12.4%
Civil servant	5.9%	3.4%
Managers in enterprise	13.1%	13.9%
Employees in enterprise	45.1%	42.1%
Doctor/Lawyer/Teacher/Journalist	8.9%	11.8%
Freelancer	16.0%	14.8%
Others	1.8%	1.6%

## Results

### Exploratory factor analysis (EFA)

The usual tests were applied to the data to assess their suitability for factor analysis (i.e., Harman’s one-factor test, item-to-total correlation analysis, Kaiser-Meyer-Olkin test, and Bartlett’s test of sphericity), and the values were all within the acceptable ranges. The first factor in principal component factor analysis accounted for 36.987% of the variance before rotation, which was less than the criterion of 40% [56]. One item was deleted for which the item-to-total correlation was below the threshold of acceptability of .3 [57]. the Kaiser-Meyer-Olkin (KMO) value was .932 and Bartlett’s test of sphericity was statistically significant (***p*** < .001). Using varimax rotation, EFA was performed on the 21 items and nine items were deleted as they either had a factor loading more than.4 on more than one factor or a factor loading less than.6 on each factor [58].

A four-dimensional 12-item scale emerged from the EFA that explained 65.4% of the variance. As shown in Table 3, the Cronbach’s alpha coefficient of the total scale was.847, and for each factor, the Cronbach’s alpha coefficients ranged from.692 to.764. The composite reliability (CR) values of the four factors fell between.749 and.805, exceeding the minimum standard of.60 suggested by Bagozzi and Yi [59] and the ideal standard of.70 suggested by Fornell and Larcker [60]. Thus, the EFA results satisfied the requirement for the reliability coefficient of the measurement scales, revealing high internal consistency. The factor loadings of each item were from.652 to.802, exceeding the minimum level of.6 suggested by Hair et al. [58]. Convergent validity was assessed by average variance extracted (AVE) for each factor. The AVE value of each factor was between.500 and.599, exceeding the cut-off value of.50, which suggested that the scale had substantial convergent validity.

**Table 3:** Exploratory factor analysis results (N = 337).

Factors	Items		Factor Loading	Cronbach’s α	CR	AVE
Transmission	tran1	The ICH is well protected.	.664	.701	.757	.501
	tran2	The ICH is transmitted from generation to generation.	.753			
	tran3	The process of transmission of the ICH is clear.	.722			
Localization	loca1	The ICH reflects the local lifestyle.	.745	.692	.749	.599
	loca2	The ICH is closely related to the local environment.	.802			
Vitality	vita1	The ICH has a special artistic style.	.711	.736	.805	.579
	vita2	The ICH encompasses a variety of forms of expression.	.799			
	vita3	The ICH is aesthetically enjoyable.	.770			
Association	asso1	I interacted with the inheritors (performers) of the ICH.	.787	.764	.794	.500
	asso2	The ICH is related to my life.	.707			
	asso3	The ICH makes me feel a sense of connection with the relevant history.	.652			
	asso4	The ICH makes me feel a sense of connection with human civilization.	.671			

### Confirmation factor analysis (CFA)

We used AMOS to test the goodness of fit between the theoretical model of ICH image derived from the EFA (four-factor model) and the data actually observed for CFA. We allowed the correlations between the factors to be freely estimated. We applied the usual tests of goodness of fit (i.e., ratio of chi-square to degrees of freedom χ^**2**^/df = 2.348, goodness-of-fit index GFI = .950, adjusted goodness-of-fit index AGFI = .919, noncomparative fit index NFI = .925, parsimonious normed fit index PNFI = .673, parsimonious goodness-of-fit PGFI = .695, and error of approximation RMSEA = 0.06), and all of the values were within the recommended ranges. Therefore, we can conclude that there was a good fit between the four-factor model of ICH image and the observed data.

To better evaluate the external quality of the four-factor model, we put forward three competitive models: a one-factor, two-factor, and a three-factor model. The results for the main indicators of a good fit of the four models are presented in Table 4. The absolute and incremental fit levels of the four-factor model were superior to those of the competitive models; thus, the four-factor model is the optimal model.

**Table 4:** Comparison of indicators of a good fit among competitive models (N = 380).

	χ^2^/df	GFI	AGFI	NFI	RMSEA	PNFI	PGFI
One-factor model	5.345	.870	.813	.808	.107	.661	.685
Two-factor model	3.296	.918	.879	.884	.078	.710	.735
Three-factor model	3.294	.933	.898	.888	.078	.686	.710
Four-factor model	2.348	.950	.919	.925	.06	.673	.695

According to Hair et al. [58], besides the goodness of fit of the measurement model, the validity of the measurement model also depends on concrete evidence of construct validity. The Cronbach’s alpha coefficient of the overall scale was.863, and those for each factor ranged from.697 to.762. The CR values of the four factors fell between.705 and.853, which was higher than the ideal standard of.70 [60]. Thus, the internal consistency of the scale is good. The factor loadings of each item were all above.6, which is higher than the minimum level of acceptability [58]. The AVE value of each factor was between.545 and.659, exceeding the cut-off value of.50, which suggests that the scale has substantial convergent validity. Because the intercorrelations between latent variables were significant but less than.8, and they were all less than the square root of AVE values of latent variables [60], we suggested that the four-factor model had very favorable discriminant validity (see Table 5).

**Table 5:** Results of the discriminant validity test (N = 380).

Latent Variables	1	2	3	4
1 Transmission	.789			
2 Localization	.610**	.738		
3 Vitality	.555**	.496**	.812	
4 Association	.487**	.483**	.436**	.766

Note: the clinodiagonal data are the square roots of AVE values of latent variables; the data below the clinodiagonal are the correlation coefficients between latent variables

**, *p* <0.01.

### Second-order measurement model for ICH image

**Fig 1:**
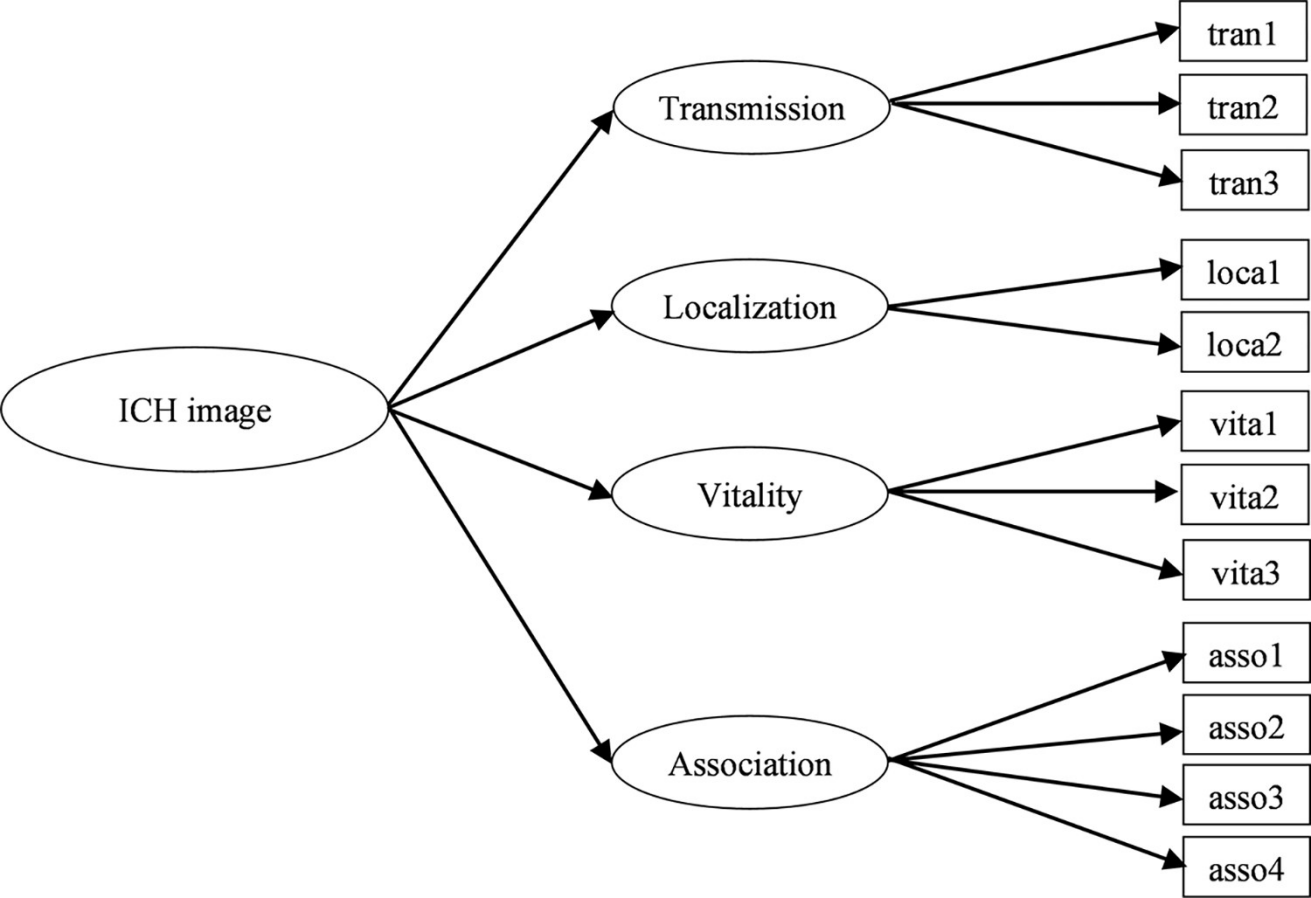
The second-order structure of ICH image.

In order to optimize the measurement model of ICH image, we assume the ICH image is a second-order reflective construct comprising four first-order factors, which are transmission, localization, vitality, and association (see Fig 1).

We used AMOS to test the second-order measurement model. We applied the usual tests for goodness of fit and discriminant standard (i.e., we determined χ^2^/df = 2.303, GFI = .949, AGFI = .920, NFI = .923, RMSEA = .059, PNFI = .700, and PGFI = .723), and all of the values were within the recommended ranges, indicating a good fit between the second-order measurement model and the data actually observed. As illustrated in Table 6, the factor loadings of each item for the first-order factors were all above.6, and the factor loadings of the four dimensions of ICH image were all above.7 and significant. Moreover, the AVE value of ICH image was.736, exceeding the cut-off value of.50, indicating substantial convergent validity; and the CR value of ICH image was.917, which is higher than the ideal standard of.70 [60], supporting internal consistency and indicating that the first-order factors well measured the second-order factor.

**Table 6:** CFA analysis for the second-order construct (N = 380).

Items	Reflective	Factors/Construct	Factor loading	T value	Sig.
tran1	←	Transmission	.633		
tran2	←	Transmission	.639	9.792	***
tran3	←	Transmission	.702	10.646	***
loca1	←	Localization	.684		
loca2	←	Localization	.696	10.773	***
vita1	←	Vitality	.761		
vita2	←	Vitality	.679	11.216	***
vita3	←	Vitality	.664	11.325	***
asso1	←	Association	.606		
asso2	←	Association	.632	9.584	***
asso3	←	Association	.713	10.017	***
asso4	←	Association	.721	9.627	***
Transmission	←	ICH image	.959		
Localization	←	ICH image	.937	9.434	***
Vitality	←	ICH image	.788	9.235	***
Association	←	ICH image	.725	7.778	***

Note:

***, *p* <0.001.

As assessing factorial invariance is necessary in the validation process, multigroup invariance analysis was chosen to test for measurement and structural invariance between different age groups (25 and below vs. 26–35, because of the sample size limit), and males and females. According to the criteria [58], the second-order measurement model fits for each group were all higher than the required value; therefore, configural invariance was established. Between different age groups, when assuming the unconstrained model is correct, the significance results of the model comparisons were all higher than 0.05 (measurement weights model, *p* = 0.810; structural weights model, *p* = 0.899; structural covariances model, *p* = 0.904; structural residuals model, *p* = 0.904). Thus, the test of measurement and structural invariance between the two age groups was supported [61–63]. Meanwhile, between the different gender groups, when assuming that the unconstrained model is correct, the significance results of model comparisons were all higher than 0.05 (measurement weights model, *p* = 0.284; structural weights model, *p* = 0.115; structural covariances model, *p* = 0.100; structural residuals model, *p* = 0.236). These findings imply that there is good support for measurement and structural invariance across genders [61–63]. The results of measurement and structural invariance can be taken as an indication of the robustness of this second-order measurement model for ICH image.

### Verification of nomological validity

We further tested the nomological validity of the measurement scale. Nomological validity analysis tests the ability of the scale to behave in accordance with a theoretical framework to link with other constructs [59, 64]. Because the link between destination image and behavioral intentions has been well established in the tourism literature [65], we chose tourist revisit intention as the nomological construct of ICH image. According to our EFA analysis and CFA analysis, ICH image consists of 12 items, which are related to the following four dimensions:

1. Transmission: The process of passing an ICH from one generation to the next within a community.
2. Localization: The ICH belongs to or is connected with the particular place or area where the community lives.
3. Vitality: The energetic and dynamic style of an ICH.
4. Association: A mental connection or relationship between tourists and the ICH/the idea suggested by the ICH.

Tourist revisit intention refers to individuals’ readiness and willingness to visit somewhere again [66]. Previous studies have suggested that the positive impact of destination image on revisit intention is due to a good image representing high perceived quality [67] and a favorable image of a specific place reinforcing travelers’ preferences for that place [68]. A positive image reflects tourists’ high evaluation of a destination where they experienced satisfaction, which will facilitate their next visit [43, 69]. Moreover, heritage tourism studies reveal that destination image is positively correlated with tourist revisit intention [70], and that heritage brand image also has a positive impact on tourists’ revisit intention [71]. Therefore, we suggest that ICH image has a positive impact on tourist revisit intention. As ICH image is a multidimensional construct, we further suggest that each dimension of ICH image has a positive impact on tourist revisit intention. The details are as follows:

In the heritage tourism literature, the quality of heritage protection is related to tourist revisit intention via pathways such as the positive relationship between object-based authenticity (which is usually concerned with high protection levels) and tourist loyalty [50]. Transmission protects the ICH and endows it with the quality of being in a living state [18], making it attractive to tourists to visit again. As many experiential factors have been found to be important influencers of tourists’ attitudes [72], and attitudes are a direct predictor of behavior intention [73], we suggest that localization and vitality, which can be considered to focus on tourists’ ICH experience, have a positive impact on tourist revisit intention. For localization, the previous sense of connection between identity and local place vanishes [74], while the localization of ICH can provide tourists with a sense and experience of a place that is valuable and attractive in the increasingly globalized world. For vitality, it contributes to ICH liveliness [75], and energetic and dynamic styles of ICH can give tourists diversified experiences, boosting the wider touristic attractiveness of the ICH. Previous studies have found that a high level of interaction with the destination by tourists can result in high levels of satisfaction and feelings of association, which then enhances tourist loyalty [72]. Association has its roots in interaction and makes tourists feel a sense of inner connection with the ICH. This mental connection works as a predictor of subsequent loyal actions.

Therefore, the following hypotheses are proposed:

H1: ICH image has a positive impact on tourist revisit intention.
H1a: Transmission has a positive impact on tourist revisit intention.
H1b: Localization has a positive impact on tourist revisit intention.
H1c: Vitality has a positive impact on tourist revisit intention.
H1d: Association has a positive impact on tourist revisit intention.

**Fig 2:**
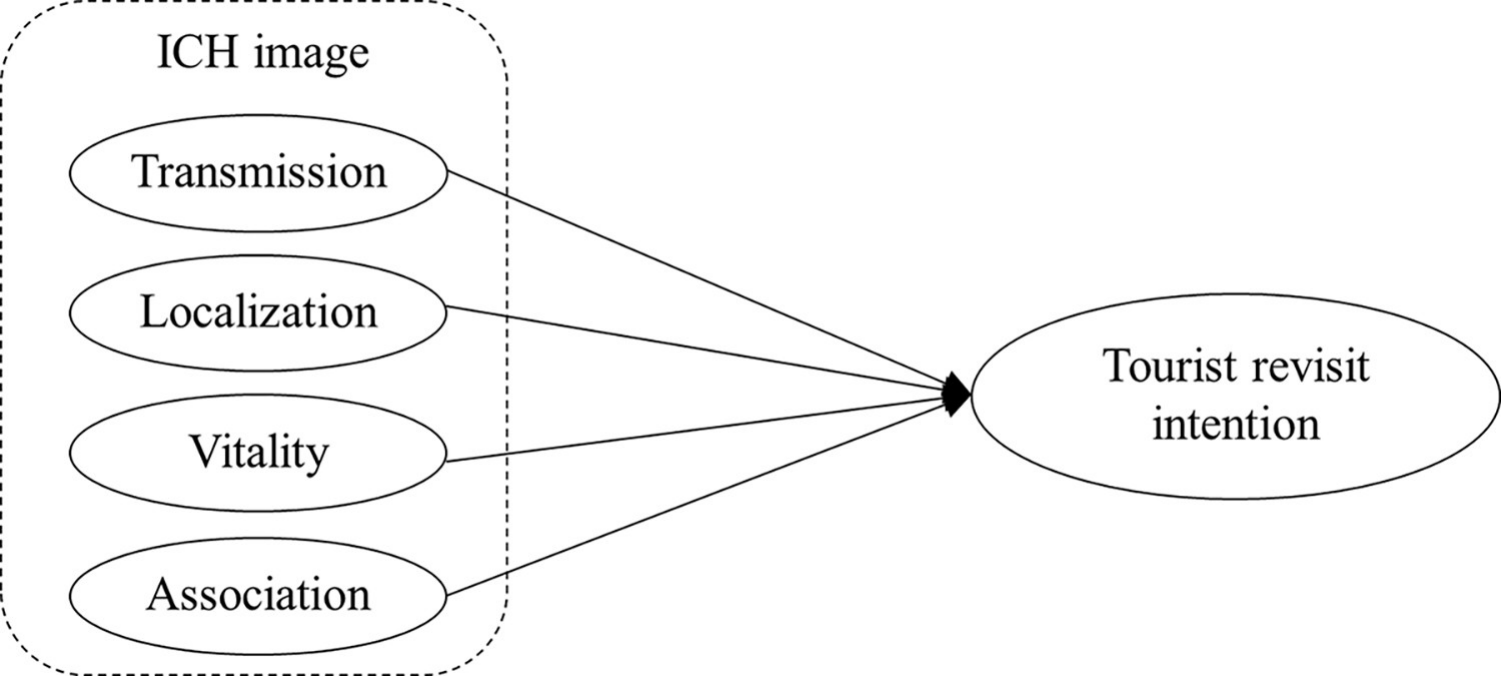
The model of nomological validity test.

The structural model with path estimates is shown in Fig 2. We used the data from Survey 2 to test the hypotheses. The measurement of tourist revisit intention was adopted from Kim’s research [54], and the Cronbach’s α in our study was.750. Hierarchical multiple regression analysis was used to test the hypotheses. We conducted a variance inflation factor (VIF) analysis to check for multicollinearity. We found that the VIF of the variables are from 1.458 to 1.919 in different regression models, which are less than the threshold value of 10. Therefore, there is no severe multicollinearity problem.

The results of the hypothesis tests are shown in Table 7. H1 predicts that ICH image will have a positive effect on tourist revisit intention. As shown in Table 7, ICH image is found to be positively related to tourist revisit intention (βimage = .673, p < 0.001). Thus, H1 is supported. In the regression model, all four dimensions of ICH image, except for localization, demonstrated significant effects on tourist revisit intention. Among the three dimensions that showed significant relationships, the transmission, vitality, and association of ICH image significantly predicted tourist revisit intention (βtran = .119, *p* < 0.05; βvita = .308, *p* < 0.001; βasso = .356, *p* < 0.001). However, the positive impact of localization of ICH image was not significant. Therefore, H1a, H1c, and H1d are supported. We suggest that this may be because of tourists’ novelty-seeking motivation [36, 76], especially in the case of the tourist gaze in cultural heritage tourism [77]. The primary purposes of cultural heritage tourism include gaining knowledge and appreciating local art, architecture, and traditions [33]. Many tourists would like to gaze at and experience new cultures but not ones they have already visited [76, 77]. Localization of the ICH image will make tourists confirm the value of the ICH they have visited, but the transmission, vitality, and association of the ICH image would mean that the ICH is likely to incorporate some new content, making it worthy of visiting again. Given the significant relationship between ICH image and the variable criterion (tourist revisit intention), as well as the relations between the three dimensions of the ICH image scale and tourist revisit intention, we conclude that the ICH image scale has demonstrated sufficient evidence of nomological validity.

**Table 7:** Results of the nomological validity test (N = 380).

Number	Hypothesis	Forecast	Standardized β	T value	Results
H1	ICH image → revisit intention	positive	.673***	17.701	supported
H1a	Transmission → revisit intention	positive	.119*	2.280	supported
H1b	Localization → revisit intention	positive	.063	1.259	not supported
H1c	Vitality → revisit intention	positive	.308***	6.496	supported
H1d	Association → revisit intention	positive	.356***	7.818	supported

Note:

*, *p* <0.05

***, *p* <0.001.

## Conclusion

ICH is of increasing interest in heritage tourism research, and researchers recognize the importance of evaluating the subjective cognition of ICH. However, research on tourist perceptions of ICH has been insufficient. As the key concept “authenticity” used in tangible cultural heritage research is not appropriate for ICH research [9, 34], we identified the concept of ICH image and developed a measurement scale for ICH image. The primary goal of the study was to test the multidimensional nature of ICH image and develop a measurement scale to facilitate assessments of tourists’ perceptions of ICH. Based on both quantitative and qualitative methods, we developed a 12-item ICH image scale comprising four first-order factors of transmission, localization, vitality, and association. Moreover, according to the nomological validity test, the relationships between the individual dimensions of ICH image and tourist revisit intention are demonstrated. Tourist loyalty, which is usually represented by revisit intention, plays a crucial role in tourism development. This study reveals that ICH image positively influences tourist revisit intention, and that the transmission vitality and association subdimensions of ICH image have a positive impact on tourist revisit intention.

### Theoretical implications

This study contributes to the literature from three aspects:

First, in the ICH management literature, the question of how tourists’ perceptions can be analyzed to help balance tourism development and the preservation of ICH in a sustainable manner has become a research focus [10]. This paper identifies the concept of ICH image, which can accurately depict tourists’ perceptions of ICH, as the commonly used concept of authenticity is not appropriate for ICH perception studies. Furthermore, we not only recognize the connotations of ICH image but also reveal the four-dimensional structure of this concept, as well as a 12-item scale for measuring it. These findings fill the gap in the literature by advancing our understanding of how tourists perceive ICH, thus helping safeguard ICH during the process of tourism development and avoiding over-commodification.

Second, this study reinforces the importance of conducting context-specific image studies [14]. We find that the extant heritage image scales or destination image scales are not suitable for measuring ICH image because those scales contain too many attributes that are unrelated to ICH. Therefore, this study develops a context-specific ICH image measurement scale that manifests the special characteristics of ICH, such as being community-based, inclusive, and “living” [1, 17–19]. Meanwhile, following the cognitive approach in image studies, the attributes of ICH are well captured by the four dimensions of ICH image.

Third, this study contributes to the heritage tourism literature by demonstrating the positive impact of ICH image on tourist revisit intention, which explains why maintaining a favorable ICH image is important for safeguarding and sustainably developing ICH within the tourism industry from a tourist behavior perspective. We also test the varying impact of each dimension of ICH image, and the significant effects of transmission, vitality, and association on tourist revisit intention are confirmed. Therefore, given the relative lack of research on tourists’ subjective cognition of ICH, this in-depth knowledge of the relationship between ICH image and tourist revisit intention should enhance our understanding of tourist behavior in ICH-based tourism.

### Practical implications

International organizations recognize the importance of safeguarding ICH, which is at risk of deterioration and degradation due to globalization and social transformation; however, the “intangible” characteristics of ICH make safeguarding operations difficult. Reliable instruments are needed for adequate management of ICH from a tourist perception perspective. Therefore, we developed an effective measurement scale to explore tourists’ perceived images of ICH. This ICH image measurement scale can be used to evaluate the level of development and protection of ICH, as a favorable ICH image will attract new tourists and re-visitors, while an unfavorable ICH image—induced by over-commercialization of the ICH—will result in the loss of the tourism market. An attractive ICH image not only means that the ICH is worth visiting for the tourist but also indicates that a fairly good balance between ICH protection and tourism development is being struck.

As the importance of preserving ICH in a sustainable manner is generally accepted by international society, balancing the preservation and development of ICH has become the responsibility of ICH management organizations. In the first stage, they can use the ICH image measurement scale to gain a full understanding of individuals’ perceptions of ICH, whether they come from outside the ICH-rooted community (tourists) or live within the community (residents), to investigate what constitutes the fragile balance. In the following stage, the double-edged sword effect raised by UNESCO’s World Heritage List [6] and operational problems can be solved in terms of four concrete aspects.

The first priority is to maintain transmission of the ICH, as if the number of successors or degree of training is insufficient, this will lead to the ICH not being properly transmitted, eventually causing it to vanish. The transmission of ICH ensures that the heritage tourism experiences are valuable. The second is to guarantee localization. As the definition of ICH emphasizes the recognition of ICH on the local level, practices, representations, expressions, knowledge, and skills that can invoke a sense of place, locale, or location will differentiate themselves from global ones and gain sustainability. Localization will help tourists understand local lifestyle and gain unique local experience in the process of globalization. The third is to gain vitality. Unlike tangible cultural heritage, ICH is “living” and constantly re-created. Thus, vitality promotes patterns of manifestation dynamically arising and giving tourists various types of enjoyment. There are many measures that can be taken to enhance vitality, such as financial support, across-culture communication, and encouraging creativity. Finally, it is important to establish associations, as close and intimate relationships reinforce an individual’s sense of identification with the ICH and strengthen its attractiveness to the world. Associations can be strengthened by tourists’ interactive experiences with ICH inheritors and per-formers during heritage tourism.

### Limitations and future research

Despite this study’s valuable contributions, it also has certain limitations. First, we still need to explore why the impact of localization on tourist revisit intention is not significant. Second, we used online sampling, and future studies may be able to conduct richer and wider sampling using other sources of respondent, such as community residents. Third, we mainly investigated tourists’ intentions rather than their behaviors, which may have more practical significance. Finally, various new technologies, such as augmented reality (AR) and virtual reality (VR), promise to change the way in which ICH is performed and displayed for tourists. Therefore, whether ICH image may also change accordingly is a question that awaits future study.

## Supporting information

S1 Data (ZIP)
