# Supplementary material for: Measuring intangible cultural heritage image: A scale development
Source: PLoS One. 2024 Jun 3;19(6):e0299088. doi: 10.1371/journal.pone.0299088 (PMC11146741; doi:10.1371/journal.pone.0299088)
Supplement: S1 Data — (ZIP) [file pone.0299088.s001.zip › S1 Data/Data in Brief - CFA 380.docx]

Survey 2

|  | | Tran1 | Tran2 | Tran3 | Loca1 | Loca2 | Vita1 | Vita2 | Vita3 |
| --- | --- | --- | --- | --- | --- | --- | --- | --- | --- |
| N | Valid | 380 | 380 | 380 | 380 | 380 | 380 | 380 | 380 |
|  | Missing | 0 | 0 | 0 | 0 | 0 | 0 | 0 | 0 |
| Mean value | | 3.08 | 2.39 | 2.66 | 2.48 | 2.67 | 2.21 | 2.22 | 2.20 |
| Median | | 3.00 | 2.00 | 2.00 | 2.00 | 2.00 | 2.00 | 2.00 | 2.00 |
| SD | | 1.429 | 1.227 | 1.313 | 1.302 | 1.355 | 1.266 | 1.214 | 1.068 |
| Variance | | 2.041 | 1.505 | 1.724 | 1.696 | 1.835 | 1.602 | 1.475 | 1.140 |
| Minimum value | | 1 | 1 | 1 | 1 | 1 | 1 | 1 | 1 |
| Maximum value | | 7 | 7 | 7 | 7 | 7 | 7 | 7 | 7 |

|  | | Asso1 | Asso2 | Asso3 | Asso4 | Revi1 | Revi2 | Revi3 |
| --- | --- | --- | --- | --- | --- | --- | --- | --- |
| N | Valid | 380 | 380 | 380 | 380 | 380 | 380 | 380 |
|  | Missing | 0 | 0 | 0 | 0 | 0 | 0 | 0 |
| Mean value | | 2.98 | 3.05 | 2.73 | 2.69 | 2.44 | 2.45 | 2.64 |
| Median | | 3.00 | 3.00 | 2.00 | 2.00 | 2.00 | 2.00 | 2.00 |
| SD | | 1.586 | 1.512 | 1.376 | 1.360 | 1.287 | 1.295 | 1.341 |
| Variance | | 2.514 | 2.285 | 1.893 | 1.850 | 1.656 | 1.678 | 1.798 |
| Minimum value | | 1 | 1 | 1 | 1 | 1 | 1 | 1 |
| Maximum value | | 7 | 7 | 7 | 7 | 7 | 7 | 7 |

| **Tran1** | | | | | |
| --- | --- | --- | --- | --- | --- |
|  | | Number | Percentage | Valid Percentage | Cumulative Percentage |
| Valid | 1 | 54 | 14.2 | 14.2 | 14.2 |
|  | 2 | 87 | 22.9 | 22.9 | 37.1 |
|  | 3 | 100 | 26.3 | 26.3 | 63.4 |
|  | 4 | 84 | 22.1 | 22.1 | 85.5 |
|  | 5 | 31 | 8.2 | 8.2 | 93.7 |
|  | 6 | 17 | 4.5 | 4.5 | 98.2 |
|  | 7 | 7 | 1.8 | 1.8 | 100.0 |
|  | Total | 380 | 100.0 | 100.0 |  |

| **Tran2** | | | | | |
| --- | --- | --- | --- | --- | --- |
|  | | Number | Percentage | Valid Percentage | Cumulative Percentage |
| Valid | 1 | 87 | 22.9 | 22.9 | 22.9 |
|  | 2 | 159 | 41.8 | 41.8 | 64.7 |
|  | 3 | 71 | 18.7 | 18.7 | 83.4 |
|  | 4 | 37 | 9.7 | 9.7 | 93.2 |
|  | 5 | 16 | 4.2 | 4.2 | 97.4 |
|  | 6 | 8 | 2.1 | 2.1 | 99.5 |
|  | 7 | 2 | .5 | .5 | 100.0 |
|  | Total | 380 | 100.0 | 100.0 |  |

| **Tran3** | | | | | |
| --- | --- | --- | --- | --- | --- |
|  | | Number | Percentage | Valid Percentage | Cumulative Percentage |
| Valid | 1 | 71 | 18.7 | 18.7 | 18.7 |
|  | 2 | 125 | 32.9 | 32.9 | 51.6 |
|  | 3 | 97 | 25.5 | 25.5 | 77.1 |
|  | 4 | 57 | 15.0 | 15.0 | 92.1 |
|  | 5 | 16 | 4.2 | 4.2 | 96.3 |
|  | 6 | 8 | 2.1 | 2.1 | 98.4 |
|  | 7 | 6 | 1.6 | 1.6 | 100.0 |
|  | Total | 380 | 100.0 | 100.0 |  |

| **Loca1** | | | | | |
| --- | --- | --- | --- | --- | --- |
|  | | Number | Percentage | Valid Percentage | Cumulative Percentage |
| Valid | 1 | 82 | 21.6 | 21.6 | 21.6 |
|  | 2 | 154 | 40.5 | 40.5 | 62.1 |
|  | 3 | 72 | 18.9 | 18.9 | 81.1 |
|  | 4 | 41 | 10.8 | 10.8 | 91.8 |
|  | 5 | 17 | 4.5 | 4.5 | 96.3 |
|  | 6 | 10 | 2.6 | 2.6 | 98.9 |
|  | 7 | 4 | 1.1 | 1.1 | 100.0 |
|  | Total | 380 | 100.0 | 100.0 |  |

| **Loca2** | | | | | |
| --- | --- | --- | --- | --- | --- |
|  | | Number | Percentage | Valid Percentage | Cumulative Percentage |
| Valid | 1 | 75 | 19.7 | 19.7 | 19.7 |
|  | 2 | 124 | 32.6 | 32.6 | 52.4 |
|  | 3 | 88 | 23.2 | 23.2 | 75.5 |
|  | 4 | 55 | 14.5 | 14.5 | 90.0 |
|  | 5 | 24 | 6.3 | 6.3 | 96.3 |
|  | 6 | 9 | 2.4 | 2.4 | 98.7 |
|  | 7 | 5 | 1.3 | 1.3 | 100.0 |
|  | Total | 380 | 100.0 | 100.0 |  |

| **Vita1** | | | | | |
| --- | --- | --- | --- | --- | --- |
|  | | Number | Percentage | Valid Percentage | Cumulative Percentage |
| Valid | 1 | 129 | 33.9 | 33.9 | 33.9 |
|  | 2 | 129 | 33.9 | 33.9 | 67.9 |
|  | 3 | 73 | 19.2 | 19.2 | 87.1 |
|  | 4 | 25 | 6.6 | 6.6 | 93.7 |
|  | 5 | 14 | 3.7 | 3.7 | 97.4 |
|  | 6 | 6 | 1.6 | 1.6 | 98.9 |
|  | 7 | 4 | 1.1 | 1.1 | 100.0 |
|  | Total | 380 | 100.0 | 100.0 |  |

| **Vita2** | | | | | |
| --- | --- | --- | --- | --- | --- |
|  | | Number | Percentage | Valid Percentage | Cumulative Percentage |
| Valid | 1 | 119 | 31.3 | 31.3 | 31.3 |
|  | 2 | 132 | 34.7 | 34.7 | 66.1 |
|  | 3 | 91 | 23.9 | 23.9 | 90.0 |
|  | 4 | 19 | 5.0 | 5.0 | 95.0 |
|  | 5 | 7 | 1.8 | 1.8 | 96.8 |
|  | 6 | 8 | 2.1 | 2.1 | 98.9 |
|  | 7 | 4 | 1.1 | 1.1 | 100.0 |
|  | Total | 380 | 100.0 | 100.0 |  |

| **Vita3** | | | | | |
| --- | --- | --- | --- | --- | --- |
|  | | Number | Percentage | Valid Percentage | Cumulative Percentage |
| Valid | 1 | 99 | 26.1 | 26.1 | 26.1 |
|  | 2 | 165 | 43.4 | 43.4 | 69.5 |
|  | 3 | 78 | 20.5 | 20.5 | 90.0 |
|  | 4 | 25 | 6.6 | 6.6 | 96.6 |
|  | 5 | 8 | 2.1 | 2.1 | 98.7 |
|  | 6 | 3 | .8 | .8 | 99.5 |
|  | 7 | 2 | .5 | .5 | 100.0 |
|  | Total | 380 | 100.0 | 100.0 |  |

| **Asso1** | | | | | |
| --- | --- | --- | --- | --- | --- |
|  | | Number | Percentage | Valid Percentage | Cumulative Percentage |
| Valid | 1 | 65 | 17.1 | 17.1 | 17.1 |
|  | 2 | 114 | 30.0 | 30.0 | 47.1 |
|  | 3 | 76 | 20.0 | 20.0 | 67.1 |
|  | 4 | 60 | 15.8 | 15.8 | 82.9 |
|  | 5 | 30 | 7.9 | 7.9 | 90.8 |
|  | 6 | 23 | 6.1 | 6.1 | 96.8 |
|  | 7 | 12 | 3.2 | 3.2 | 100.0 |
|  | Total | 380 | 100.0 | 100.0 |  |

| **Asso2** | | | | | |
| --- | --- | --- | --- | --- | --- |
|  | | Number | Percentage | Valid Percentage | Cumulative Percentage |
| Valid | 1 | 62 | 16.3 | 16.3 | 16.3 |
|  | 2 | 89 | 23.4 | 23.4 | 39.7 |
|  | 3 | 96 | 25.3 | 25.3 | 65.0 |
|  | 4 | 73 | 19.2 | 19.2 | 84.2 |
|  | 5 | 32 | 8.4 | 8.4 | 92.6 |
|  | 6 | 17 | 4.5 | 4.5 | 97.1 |
|  | 7 | 11 | 2.9 | 2.9 | 100.0 |
|  | Total | 380 | 100.0 | 100.0 |  |

| **Asso3** | | | | | |
| --- | --- | --- | --- | --- | --- |
|  | | Number | Percentage | Valid Percentage | Cumulative Percentage |
| Valid | 1 | 59 | 15.5 | 15.5 | 15.5 |
|  | 2 | 140 | 36.8 | 36.8 | 52.4 |
|  | 3 | 98 | 25.8 | 25.8 | 78.2 |
|  | 4 | 42 | 11.1 | 11.1 | 89.2 |
|  | 5 | 18 | 4.7 | 4.7 | 93.9 |
|  | 6 | 16 | 4.2 | 4.2 | 98.2 |
|  | 7 | 7 | 1.8 | 1.8 | 100.0 |
|  | Total | 380 | 100.0 | 100.0 |  |

| **Asso4** | | | | | |
| --- | --- | --- | --- | --- | --- |
|  | | Number | Percentage | Valid Percentage | Cumulative Percentage |
| Valid | 1 | 73 | 19.2 | 19.2 | 19.2 |
|  | 2 | 124 | 32.6 | 32.6 | 51.8 |
|  | 3 | 88 | 23.2 | 23.2 | 75.0 |
|  | 4 | 58 | 15.3 | 15.3 | 90.3 |
|  | 5 | 21 | 5.5 | 5.5 | 95.8 |
|  | 6 | 11 | 2.9 | 2.9 | 98.7 |
|  | 7 | 5 | 1.3 | 1.3 | 100.0 |
|  | Total | 380 | 100.0 | 100.0 |  |

| **Revi1** | | | | | |
| --- | --- | --- | --- | --- | --- |
|  | | Number | Percentage | Valid Percentage | Cumulative Percentage |
| Valid | 1 | 92 | 24.2 | 24.2 | 24.2 |
|  | 2 | 138 | 36.3 | 36.3 | 60.5 |
|  | 3 | 86 | 22.6 | 22.6 | 83.2 |
|  | 4 | 36 | 9.5 | 9.5 | 92.6 |
|  | 5 | 15 | 3.9 | 3.9 | 96.6 |
|  | 6 | 9 | 2.4 | 2.4 | 98.9 |
|  | 7 | 4 | 1.1 | 1.1 | 100.0 |
|  | Total | 380 | 100.0 | 100.0 |  |

| **Revi2** | | | | | |
| --- | --- | --- | --- | --- | --- |
|  | | Number | Percentage | Valid Percentage | Cumulative Percentage |
| Valid | 1 | 91 | 23.9 | 23.9 | 23.9 |
|  | 2 | 135 | 35.5 | 35.5 | 59.5 |
|  | 3 | 93 | 24.5 | 24.5 | 83.9 |
|  | 4 | 34 | 8.9 | 8.9 | 92.9 |
|  | 5 | 12 | 3.2 | 3.2 | 96.1 |
|  | 6 | 10 | 2.6 | 2.6 | 98.7 |
|  | 7 | 5 | 1.3 | 1.3 | 100.0 |
|  | Total | 380 | 100.0 | 100.0 |  |

| **Revi3** | | | | | |
| --- | --- | --- | --- | --- | --- |
|  | | Number | Percentage | Valid Percentage | Cumulative Percentage |
| Valid | 1 | 75 | 19.7 | 19.7 | 19.7 |
|  | 2 | 126 | 33.2 | 33.2 | 52.9 |
|  | 3 | 94 | 24.7 | 24.7 | 77.6 |
|  | 4 | 51 | 13.4 | 13.4 | 91.1 |
|  | 5 | 19 | 5.0 | 5.0 | 96.1 |
|  | 6 | 9 | 2.4 | 2.4 | 98.4 |
|  | 7 | 6 | 1.6 | 1.6 | 100.0 |
|  | Total | 380 | 100.0 | 100.0 |  |

| **Gender** | | | | | |
| --- | --- | --- | --- | --- | --- |
|  | | Number | Percentage | Valid Percentage | Cumulative percentage |
| Valid | Male | 209 | 55.0 | 55.0 | 55.0 |
|  | Female | 171 | 45.0 | 45.0 | 100.0 |
|  | Total | 380 | 100.0 | 100.0 |  |

| **Age** | | | | | |
| --- | --- | --- | --- | --- | --- |
|  | | Number | Percentage | Valid Percentage | Cumulative percentage |
| Valid | 25 and below | 104 | 27.4 | 27.4 | 27.4 |
|  | 26-35 | 178 | 46.8 | 46.8 | 74.2 |
|  | 36-45 | 71 | 18.7 | 18.7 | 92.9 |
|  | 46-55 | 26 | 6.8 | 6.8 | 99.7 |
|  | 56 and above | 1 | 0.3 | 0.3 | 100.0 |
|  | Total | 380 | 100.0 | 100.0 |  |

| **Education** | | | | | |
| --- | --- | --- | --- | --- | --- |
|  | | Number | Percentage | Valid Percentage | Cumulative percentage |
| Valid | Up to high school | 63 | 16.6 | 16.6 | 16.6 |
|  | College degree | 138 | 36.3 | 36.3 | 52.9 |
|  | Bachelor’s degree | 167 | 43.9 | 43.9 | 96.8 |
|  | Postgraduate degree and above | 12 | 3.2 | 3.2 | 100.0 |
|  | Total | 380 | 100.0 | 100.0 |  |

| **Profession** | | | | | |
| --- | --- | --- | --- | --- | --- |
|  | | Number | Percentage | Valid Percentage | Cumulative percentage |
| Valid | Student | 47 | 12.4 | 12.4 | 12.4 |
|  | Civil servant | 13 | 3.4 | 3.4 | 15.8 |
|  | Managers in enterprise | 53 | 13.9 | 13.9 | 29.7 |
|  | Employees in enterprise | 160 | 42.1 | 42.1 | 71.8 |
|  | Doctor/Lawyer/Teacher/Journalist | 45 | 11.8 | 11.8 | 83.6 |
|  | Freelancer | 56 | 14.8 | 14.8 | 98.4 |
|  | Others | 6 | 1.6 | 1.6 | 100.0 |
|  | Total | 380 | 100.0 | 100.0 |  |

| **Income per month** | | | | | |
| --- | --- | --- | --- | --- | --- |
|  | | Number | Percentage | Valid Percentage | Cumulative percentage |
| Valid | No income | 40 | 10.5 | 10.5 | 10.5 |
|  | RMB1–5,000 | 70 | 18.4 | 18.4 | 28.9 |
|  | RMB5,001–8,000 | 203 | 53.4 | 53.4 | 82.4 |
|  | RMB8,001 and above | 59 | 15.5 | 15.5 | 97.9 |
|  | Inconvenient to disclose | 8 | 2.1 | 2.1 | 100.0 |
|  | Total | 380 | 100.0 | 100.0 |  |
