# Supplementary material for: Measuring intangible cultural heritage image: A scale development
Source: PLoS One. 2024 Jun 3;19(6):e0299088. doi: 10.1371/journal.pone.0299088 (PMC11146741; doi:10.1371/journal.pone.0299088)
Supplement: S1 Data — (ZIP) [file pone.0299088.s001.zip › S1 Data/Data in Brief - EFA 337.docx]

**Survey 1**

|  | | Item1 | Item2 | Item3 | Item4 | Item5 | Item6 | Item7 | Item8 | Item9 | Item10 | Item11 |
| --- | --- | --- | --- | --- | --- | --- | --- | --- | --- | --- | --- | --- |
| N | Valid | 337 | 337 | 337 | 337 | 337 | 337 | 337 | 337 | 337 | 337 | 337 |
|  | Missing | 0 | 0 | 0 | 0 | 0 | 0 | 0 | 0 | 0 | 0 | 0 |
| Mean value | | 1.93 | 2.38 | 3.20 | 2.40 | 2.66 | 2.39 | 2.51 | 2.20 | 2.61 | 2.32 | 2.21 |
| Median | | 2.00 | 2.00 | 3.00 | 2.00 | 2.00 | 2.00 | 2.00 | 2.00 | 2.00 | 2.00 | 2.00 |
| SD | | 1.059 | 1.161 | 1.367 | 1.283 | 1.311 | 1.396 | 1.350 | 1.143 | 1.344 | 1.320 | 1.241 |
| Variance | | 1.121 | 1.349 | 1.870 | 1.645 | 1.718 | 1.948 | 1.822 | 1.307 | 1.805 | 1.742 | 1.540 |
| Minimum value | | 1 | 1 | 1 | 1 | 1 | 1 | 1 | 1 | 1 | 1 | 1 |
| Maximum value | | 7 | 7 | 7 | 7 | 7 | 7 | 7 | 7 | 7 | 7 | 7 |

|  | | Item12 | Item13 | Item14 | Item15 | Item16 | Item17 | Item18 | Item19 | Item20 | Item21 | Item22 |
| --- | --- | --- | --- | --- | --- | --- | --- | --- | --- | --- | --- | --- |
| N | Valid | 337 | 337 | 337 | 337 | 337 | 337 | 337 | 337 | 337 | 337 | 337 |
|  | Missing | 0 | 0 | 0 | 0 | 0 | 0 | 0 | 0 | 0 | 0 | 0 |
| Mean value | | 2.27 | 2.03 | 2.20 | 2.36 | 2.58 | 2.75 | 3.58 | 2.96 | 3.06 | 2.77 | 2.65 |
| Median | | 2.00 | 2.00 | 2.00 | 2.00 | 2.00 | 2.00 | 3.00 | 3.00 | 3.00 | 2.00 | 2.00 |
| SD | | 1.240 | 1.100 | 1.112 | 1.291 | 1.365 | 1.398 | 1.921 | 1.491 | 1.553 | 1.421 | 1.379 |
| Variance | | 1.538 | 1.210 | 1.237 | 1.666 | 1.863 | 1.954 | 3.690 | 2.222 | 2.411 | 2.019 | 1.901 |
| Minimum value | | 1 | 1 | 1 | 1 | 1 | 1 | 1 | 1 | 1 | 1 | 1 |
| Maximum value | | 7 | 7 | 7 | 6 | 7 | 7 | 7 | 7 | 7 | 7 | 7 |

| **Item1** | | | | | |
| --- | --- | --- | --- | --- | --- |
|  | | Number | Percentage | Valid Percentage | Cumulative Percentage |
| Valid | 1 | 146 | 43.3 | 43.3 | 43.3 |
|  | 2 | 102 | 30.3 | 30.3 | 73.6 |
|  | 3 | 67 | 19.9 | 19.9 | 93.5 |
|  | 4 | 14 | 4.2 | 4.2 | 97.6 |
|  | 5 | 4 | 1.2 | 1.2 | 98.8 |
|  | 6 | 3 | .9 | .9 | 99.7 |
|  | 7 | 1 | .3 | .3 | 100.0 |
|  | Total | 337 | 100.0 | 100.0 |  |

| **Item2** | | | | | |
| --- | --- | --- | --- | --- | --- |
|  | | Number | Percentage | Valid Percentage | Cumulative percentage |
| Valid | 1 | 67 | 19.9 | 19.9 | 19.9 |
|  | 2 | 153 | 45.4 | 45.4 | 65.3 |
|  | 3 | 71 | 21.1 | 21.1 | 86.4 |
|  | 4 | 24 | 7.1 | 7.1 | 93.5 |
|  | 5 | 16 | 4.7 | 4.7 | 98.2 |
|  | 6 | 3 | .9 | .9 | 99.1 |
|  | 7 | 3 | .9 | .9 | 100.0 |
|  | Total | 337 | 100.0 | 100.0 |  |

| **Item3** | | | | | |
| --- | --- | --- | --- | --- | --- |
|  | | Number | Percentage | Valid Percentage | Cumulative percentage |
| Valid | 1 | 35 | 10.4 | 10.4 | 10.4 |
|  | 2 | 69 | 20.5 | 20.5 | 30.9 |
|  | 3 | 107 | 31.8 | 31.8 | 62.6 |
|  | 4 | 73 | 21.7 | 21.7 | 84.3 |
|  | 5 | 29 | 8.6 | 8.6 | 92.9 |
|  | 6 | 20 | 5.9 | 5.9 | 98.8 |
|  | 7 | 4 | 1.2 | 1.2 | 100.0 |
|  | Total | 337 | 100.0 | 100.0 |  |

| **Item4** | | | | | |  |
| --- | --- | --- | --- | --- | --- | --- |
|  | | Number | Percentage | Valid Percentage | Cumulative percentage | |
| Valid | 1 | 89 | 26.4 | 26.4 | 26.4 | |
|  | 2 | 119 | 35.3 | 35.3 | 61.7 | |
|  | 3 | 72 | 21.4 | 21.4 | 83.1 | |
|  | 4 | 33 | 9.8 | 9.8 | 92.9 | |
|  | 5 | 14 | 4.2 | 4.2 | 97.0 | |
|  | 6 | 7 | 2.1 | 2.1 | 99.1 | |
|  | 7 | 3 | .9 | .9 | 100.0 | |
|  | Total | 337 | 100.0 | 100.0 |  | |

| **Item5** | | | | | |  |
| --- | --- | --- | --- | --- | --- | --- |
|  | | Number | Percentage | Valid Percentage | Cumulative percentage | |
| Valid | 1 | 60 | 17.8 | 17.8 | 17.8 | |
|  | 2 | 125 | 37.1 | 37.1 | 54.9 | |
|  | 3 | 66 | 19.6 | 19.6 | 74.5 | |
|  | 4 | 50 | 14.8 | 14.8 | 89.3 | |
|  | 5 | 29 | 8.6 | 8.6 | 97.9 | |
|  | 6 | 4 | 1.2 | 1.2 | 99.1 | |
|  | 7 | 3 | .9 | .9 | 100.0 | |
|  | Total | 337 | 100.0 | 100.0 |  | |

| **Item6** | | | | | |  |
| --- | --- | --- | --- | --- | --- | --- |
|  | | Number | Percentage | Valid Percentage | Cumulative percentage | |
| Valid | 1 | 104 | 30.9 | 30.9 | 30.9 | |
|  | 2 | 112 | 33.2 | 33.2 | 64.1 | |
|  | 3 | 50 | 14.8 | 14.8 | 78.9 | |
|  | 4 | 45 | 13.4 | 13.4 | 92.3 | |
|  | 5 | 10 | 3.0 | 3.0 | 95.3 | |
|  | 6 | 13 | 3.9 | 3.9 | 99.1 | |
|  | 7 | 3 | .9 | .9 | 100.0 | |
|  | Total | 337 | 100.0 | 100.0 |  | |
| **Item7** | | | | | |  |
|  | | Number | Percentage | Valid Percentage | Cumulative percentage | |
| Valid | 1 | 70 | 20.8 | 20.8 | 20.8 | |
|  | 2 | 142 | 42.1 | 42.1 | 62.9 | |
|  | 3 | 62 | 18.4 | 18.4 | 81.3 | |
|  | 4 | 30 | 8.9 | 8.9 | 90.2 | |
|  | 5 | 18 | 5.3 | 5.3 | 95.5 | |
|  | 6 | 10 | 3.0 | 3.0 | 98.5 | |
|  | 7 | 5 | 1.5 | 1.5 | 100.0 | |
|  | Total | 337 | 100.0 | 100.0 |  | |

| **Item8** | | | | | |  |
| --- | --- | --- | --- | --- | --- | --- |
|  | | Number | Percentage | Valid Percentage | Cumulative percentage | |
| Valid | 1 | 96 | 28.5 | 28.5 | 28.5 | |
|  | 2 | 143 | 42.4 | 42.4 | 70.9 | |
|  | 3 | 59 | 17.5 | 17.5 | 88.4 | |
|  | 4 | 21 | 6.2 | 6.2 | 94.7 | |
|  | 5 | 12 | 3.6 | 3.6 | 98.2 | |
|  | 6 | 5 | 1.5 | 1.5 | 99.7 | |
|  | 7 | 1 | .3 | .3 | 100.0 | |
|  | Total | 337 | 100.0 | 100.0 |  | |

| **Item9** | | | | | |
| --- | --- | --- | --- | --- | --- |
|  | | Number | Percentage | Valid Percentage | Cumulative percentage |
| Valid | 1 | 66 | 19.6 | 19.6 | 19.6 |
|  | 2 | 122 | 36.2 | 36.2 | 55.8 |
|  | 3 | 82 | 24.3 | 24.3 | 80.1 |
|  | 4 | 32 | 9.5 | 9.5 | 89.6 |
|  | 5 | 19 | 5.6 | 5.6 | 95.3 |
|  | 6 | 13 | 3.9 | 3.9 | 99.1 |
|  | 7 | 3 | .9 | .9 | 100.0 |
|  | Total | 337 | 100.0 | 100.0 |  |

| **Item10** | | | | | |  |
| --- | --- | --- | --- | --- | --- | --- |
|  | | Number | Percentage | Valid Percentage | Cumulative percentage | |
| Valid | 1 | 113 | 33.5 | 33.5 | 33.5 | |
|  | 2 | 94 | 27.9 | 27.9 | 61.4 | |
|  | 3 | 76 | 22.6 | 22.6 | 84.0 | |
|  | 4 | 30 | 8.9 | 8.9 | 92.9 | |
|  | 5 | 13 | 3.9 | 3.9 | 96.7 | |
|  | 6 | 9 | 2.7 | 2.7 | 99.4 | |
|  | 7 | 2 | .6 | .6 | 100.0 | |
|  | Total | 337 | 100.0 | 100.0 |  | |

| **Item11** | | | | | |
| --- | --- | --- | --- | --- | --- |
|  | | Number | Percentage | Valid Percentage | Cumulative percentage |
| Valid | 1 | 116 | 34.4 | 34.4 | 34.4 |
|  | 2 | 109 | 32.3 | 32.3 | 66.8 |
|  | 3 | 68 | 20.2 | 20.2 | 86.9 |
|  | 4 | 23 | 6.8 | 6.8 | 93.8 |
|  | 5 | 14 | 4.2 | 4.2 | 97.9 |
|  | 6 | 5 | 1.5 | 1.5 | 99.4 |
|  | 7 | 2 | .6 | .6 | 100.0 |
|  | Total | 337 | 100.0 | 100.0 |  |

| **Item12** | | | | | |
| --- | --- | --- | --- | --- | --- |
|  | | Number | Percentage | Valid Percentage | Cumulative percentage |
| Valid | 1 | 110 | 32.6 | 32.6 | 32.6 |
|  | 2 | 101 | 30.0 | 30.0 | 62.6 |
|  | 3 | 78 | 23.1 | 23.1 | 85.8 |
|  | 4 | 29 | 8.6 | 8.6 | 94.4 |
|  | 5 | 12 | 3.6 | 3.6 | 97.9 |
|  | 6 | 5 | 1.5 | 1.5 | 99.4 |
|  | 7 | 2 | .6 | .6 | 100.0 |
|  | Total | 337 | 100.0 | 100.0 |  |

| **Item13** | | | | | |
| --- | --- | --- | --- | --- | --- |
|  | | Number | Percentage | Valid Percentage | Cumulative percentage |
| Valid | 1 | 120 | 35.6 | 35.6 | 35.6 |
|  | 2 | 135 | 40.1 | 40.1 | 75.7 |
|  | 3 | 54 | 16.0 | 16.0 | 91.7 |
|  | 4 | 14 | 4.2 | 4.2 | 95.8 |
|  | 5 | 8 | 2.4 | 2.4 | 98.2 |
|  | 6 | 5 | 1.5 | 1.5 | 99.7 |
|  | 7 | 1 | .3 | .3 | 100.0 |
|  | Total | 337 | 100.0 | 100.0 |  |

| **Item14** | | | | | |
| --- | --- | --- | --- | --- | --- |
|  | | Number | Percentage | Valid Percentage | Cumulative percentage |
| Valid | 1 | 90 | 26.7 | 26.7 | 26.7 |
|  | 2 | 150 | 44.5 | 44.5 | 71.2 |
|  | 3 | 60 | 17.8 | 17.8 | 89.0 |
|  | 4 | 20 | 5.9 | 5.9 | 95.0 |
|  | 5 | 13 | 3.9 | 3.9 | 98.8 |
|  | 6 | 2 | .6 | .6 | 99.4 |
|  | 7 | 2 | .6 | .6 | 100.0 |
|  | Total | 337 | 100.0 | 100.0 |  |

| **Item15** | | | | | |
| --- | --- | --- | --- | --- | --- |
|  | | Number | Percentage | Valid Percentage | Cumulative percentage |
| Valid | 1 | 100 | 29.7 | 29.7 | 29.7 |
|  | 2 | 107 | 31.8 | 31.8 | 61.4 |
|  | 3 | 77 | 22.8 | 22.8 | 84.3 |
|  | 4 | 24 | 7.1 | 7.1 | 91.4 |
|  | 5 | 19 | 5.6 | 5.6 | 97.0 |
|  | 6 | 10 | 3.0 | 3.0 | 100.0 |
|  | Total | 337 | 100.0 | 100.0 |  |

| **Item16** | | | | | |
| --- | --- | --- | --- | --- | --- |
|  | | Number | Percentage | Valid Percentage | Cumulative percentage |
| Valid | 1 | 72 | 21.4 | 21.4 | 21.4 |
|  | 2 | 124 | 36.8 | 36.8 | 58.2 |
|  | 3 | 64 | 19.0 | 19.0 | 77.2 |
|  | 4 | 45 | 13.4 | 13.4 | 90.5 |
|  | 5 | 19 | 5.6 | 5.6 | 96.1 |
|  | 6 | 8 | 2.4 | 2.4 | 98.5 |
|  | 7 | 5 | 1.5 | 1.5 | 100.0 |
|  | Total | 337 | 100.0 | 100.0 |  |

| **Item17** | | | | | |
| --- | --- | --- | --- | --- | --- |
|  | | Number | Percentage | Valid Percentage | Cumulative percentage |
| Valid | 1 | 63 | 18.7 | 18.7 | 18.7 |
|  | 2 | 108 | 32.0 | 32.0 | 50.7 |
|  | 3 | 70 | 20.8 | 20.8 | 71.5 |
|  | 4 | 63 | 18.7 | 18.7 | 90.2 |
|  | 5 | 17 | 5.0 | 5.0 | 95.3 |
|  | 6 | 10 | 3.0 | 3.0 | 98.2 |
|  | 7 | 6 | 1.8 | 1.8 | 100.0 |
|  | Total | 337 | 100.0 | 100.0 |  |

| **Item18** | | | | | |
| --- | --- | --- | --- | --- | --- |
|  | | Number | Percentage | Valid Percentage | Cumulative percentage |
| Valid | 1 | 51 | 15.1 | 15.1 | 15.1 |
|  | 2 | 70 | 20.8 | 20.8 | 35.9 |
|  | 3 | 69 | 20.5 | 20.5 | 56.4 |
|  | 4 | 32 | 9.5 | 9.5 | 65.9 |
|  | 5 | 35 | 10.4 | 10.4 | 76.3 |
|  | 6 | 53 | 15.7 | 15.7 | 92.0 |
|  | 7 | 27 | 8.0 | 8.0 | 100.0 |
|  | Total | 337 | 100.0 | 100.0 |  |

| **Item19** | | | | | |
| --- | --- | --- | --- | --- | --- |
|  | | Number | Percentage | Valid Percentage | Cumulative percentage |
| Valid | 1 | 52 | 15.4 | 15.4 | 15.4 |
|  | 2 | 99 | 29.4 | 29.4 | 44.8 |
|  | 3 | 83 | 24.6 | 24.6 | 69.4 |
|  | 4 | 47 | 13.9 | 13.9 | 83.4 |
|  | 5 | 32 | 9.5 | 9.5 | 92.9 |
|  | 6 | 17 | 5.0 | 5.0 | 97.9 |
|  | 7 | 7 | 2.1 | 2.1 | 100.0 |
|  | Total | 337 | 100.0 | 100.0 |  |

| **Item20** | | | | | |
| --- | --- | --- | --- | --- | --- |
|  | | Number | Percentage | Valid Percentage | Cumulative percentage |
| Valid | 1 | 46 | 13.6 | 13.6 | 13.6 |
|  | 2 | 100 | 29.7 | 29.7 | 43.3 |
|  | 3 | 84 | 24.9 | 24.9 | 68.2 |
|  | 4 | 46 | 13.6 | 13.6 | 81.9 |
|  | 5 | 29 | 8.6 | 8.6 | 90.5 |
|  | 6 | 21 | 6.2 | 6.2 | 96.7 |
|  | 7 | 11 | 3.3 | 3.3 | 100.0 |
|  | Total | 337 | 100.0 | 100.0 |  |

| **Item21** | | | | | |
| --- | --- | --- | --- | --- | --- |
|  | | Number | Percentage | Valid Percentage | Cumulative percentage |
| Valid | 1 | 58 | 17.2 | 17.2 | 17.2 |
|  | 2 | 115 | 34.1 | 34.1 | 51.3 |
|  | 3 | 83 | 24.6 | 24.6 | 76.0 |
|  | 4 | 35 | 10.4 | 10.4 | 86.4 |
|  | 5 | 24 | 7.1 | 7.1 | 93.5 |
|  | 6 | 19 | 5.6 | 5.6 | 99.1 |
|  | 7 | 3 | .9 | .9 | 100.0 |
|  | Total | 337 | 100.0 | 100.0 |  |

| **Item22** | | | | | |
| --- | --- | --- | --- | --- | --- |
|  | | Number | Percentage | Valid Percentage | Cumulative percentage |
| Valid | 1 | 69 | 20.5 | 20.5 | 20.5 |
|  | 2 | 108 | 32.0 | 32.0 | 52.5 |
|  | 3 | 88 | 26.1 | 26.1 | 78.6 |
|  | 4 | 38 | 11.3 | 11.3 | 89.9 |
|  | 5 | 18 | 5.3 | 5.3 | 95.3 |
|  | 6 | 10 | 3.0 | 3.0 | 98.2 |
|  | 7 | 6 | 1.8 | 1.8 | 100.0 |
|  | Total | 337 | 100.0 | 100.0 |  |

| **Gender** | | | | | |
| --- | --- | --- | --- | --- | --- |
|  | | Number | Percentage | Valid Percentage | Cumulative percentage |
| Valid | Male | 162 | 48.1 | 48.1 | 48.1 |
|  | Female | 175 | 51.9 | 51.9 | 100.0 |
|  | Total | 337 | 100.0 | 100.0 |  |

| **Age** | | | | | |
| --- | --- | --- | --- | --- | --- |
|  | | Number | Percentage | Valid Percentage | Cumulative percentage |
| Valid | 25 and below | 76 | 22.6 | 22.6 | 22.6 |
|  | 26-35 | 193 | 57.3 | 57.3 | 79.8 |
|  | 36-45 | 64 | 19.0 | 19.0 | 98.8 |
|  | 46-55 | 4 | 1.2 | 1.2 | 100.0 |
|  | Total | 337 | 100.0 | 100.0 |  |

| **Education** | | | | | |
| --- | --- | --- | --- | --- | --- |
|  | | Number | Percentage | Valid Percentage | Cumulative percentage |
| Valid | Up to high school | 38 | 11.3 | 11.3 | 11.3 |
|  | College degree | 92 | 27.3 | 27.3 | 38.6 |
|  | Bachelor’s degree | 202 | 59.9 | 59.9 | 98.5 |
|  | Postgraduate degree and above | 5 | 1.5 | 1.5 | 100.0 |
|  | Total | 337 | 100.0 | 100.0 |  |

| **Profession** | | | | | |
| --- | --- | --- | --- | --- | --- |
|  | | Number | Percentage | Valid Percentage | Cumulative percentage |
| Valid | Student | 31 | 9.2 | 9.2 | 9.2 |
|  | Civil servant | 20 | 5.9 | 5.9 | 15.1 |
|  | Managers in enterprise | 44 | 13.1 | 13.1 | 28.2 |
|  | Employees in enterprise | 152 | 45.1 | 45.1 | 73.3 |
|  | Doctor/Lawyer/Teacher/Journalist | 30 | 8.9 | 8.9 | 82.2 |
|  | Freelancer | 54 | 16 | 16 | 88.2 |
|  | Others | 6 | 1.8 | 1.8 | 100.0 |
|  | Total | 337 | 100.0 | 100.0 |  |

| **Income per month** | | | | | |
| --- | --- | --- | --- | --- | --- |
|  | | Number | Percentage | Valid Percentage | Cumulative percentage |
| Valid | No income | 21 | 6.2 | 6.2 | 6.2 |
|  | RMB1–5,000 | 68 | 20.2 | 20.2 | 26.4 |
|  | RMB5,001–8,000 | 196 | 58.2 | 58.2 | 84.6 |
|  | RMB8,001 and above | 47 | 14 | 14 | 98.6 |
|  | Inconvenient to disclose | 5 | 1.5 | 1.5 | 100.0 |
|  | Total | 337 | 100.0 | 100.0 |  |
